# Spontaneous conception and successful live birth during hormone replacement therapy in a patient with iatrogenic premature ovarian insufficiency: a case report

**DOI:** 10.3389/fmed.2026.1929801

**Published:** 2026-08-25

**Authors:** Jung Min Lee, Ju Seok Yang, Ji Eun Park, Jong Chul Baek, Jae Yoon Jo, Hyen Chul Jo

**Affiliations:** 1 Department of Obstetrics and Gynecology, Gyeongsang National University Changwon Hospital, Changwon, Republic of Korea; 2 Institute of Health Science, Gyeongsang National University, Jinju, Republic of Korea; 3 Department of Obstetrics and Gynecology, Gyeongsang National University School of Medicine, Jinju, Republic of Korea; 4 Department of Obstetrics and Gynecology, Gyeongsang National University Hospital, Jinju, Republic of Korea

**Keywords:** anti-Müllerian hormone, fertility preservation, hormone replacement therapy, premature ovarian insufficiency, spontaneous pregnancy

## Abstract

**Background:**

Premature ovarian insufficiency (POI) is defined as loss of ovarian function before the age of 40 years. It has a profound impact on patients’ quality of life, particularly due to infertility and menopausal symptoms related to estrogen deficiency. Although most patients diagnosed with POI require assisted reproductive technologies (ART) or oocyte donation to achieve pregnancy, a small subset experiences a spontaneous resumption of ovarian function, leading to natural conception in approximately 5 to 10% of cases. Notably, this likelihood of spontaneous pregnancy is postulated to be substantially lower in iatrogenic POI than in cases of idiopathic or spontaneous etiologies.

**Case presentation:**

We report a rare case of 37-year-old woman diagnosed with iatrogenic POI after repeated bilateral ovarian surgeries for endometriosis. Despite biochemical evidence of ovarian insufficiency, including an elevated follicle-stimulating hormone (FSH) level of 66.55 IU/L and low anti-Müllerian hormone (AMH) levels of 0.02 ng/mL, she presented with spontaneous conception during hormone replacement therapy. The pregnancy course was largely uneventful except for a transient episode of threatened abortion, which resolved with conservative management. A healthy infant was delivered at 36 weeks of gestation via cesarean section without complications.

**Conclusion:**

We report a rare case of spontaneous ovarian recovery and natural conception in a patient with iatrogenic POI following recurrent endometriosis surgeries. This case highlights the need for clinicians to recognize the potential for spontaneous pregnancy in this population and provide appropriate counseling on fertility and contraception during hormone replacement therapy.

## Introduction

Premature ovarian insufficiency (POI) is characterized by the loss of ovarian function before the age of 40 years. Diagnosis is based on menstrual cycle abnormalities persisting for at least 4 months, accompanied by an elevated follicle stimulating hormone (FSH) level equal to or greater than 25 IU/L. In cases of diagnostic uncertainty, FSH level can be retested after an interval of 4 to 6 weeks (1). The prevalence of POI is reported to be approximately 3.7%, with variations in distribution observed across different geographic regions (2). It has a detrimental impact not only on fertility but also on overall health, including increased risks of cardiovascular disease, osteoporosis, and reduced quality of life. In women with POI, hormone replacement therapy (HRT) is commonly used to alleviate menopausal symptoms and reduce long-term health risks associated with estrogen deficiency.

While the underlying pathophysiology has not been fully elucidated, POI is thought to result from diminished primordial follicle reserves, accelerated follicular atresia, impaired follicle maturation, or follicular dysfunction (3, 4).

Although the etiologies of POI are heterogeneous and include genetic, autoimmune, metabolic, infectious, environmental, and iatrogenic factors, the underlying etiology remains undetermined in the majority of cases. Among the established etiologies, iatrogenic POI accounts for approximately 50% of all POI cases (5) and may result from chemotherapy, radiotherapy, ovarian surgery, and various other medical treatments that damage ovarian function. Despite the markedly reduced fertility associated with POI, a small proportion of patients may experience spontaneous recovery of ovarian function, allowing natural conception. Previous studies have reported that approximately 5 to 10% of women with POI achieve spontaneous pregnancy (6). However, spontaneous conception appears to be considerably less common in women with iatrogenic POI than in those with idiopathic or spontaneous POI. Moreover, the available evidence remains limited because previous studies have predominantly focused on fertility outcomes following assisted reproductive technology (ART) in POI (7).

We report a rare case of a patient diagnosed with iatrogenic POI following repeated bilateral ovarian endometriosis surgeries, who achieved a spontaneous pregnancy and a successful live birth during hormone replacement therapy.

## Case presentation

**Figure 1:**
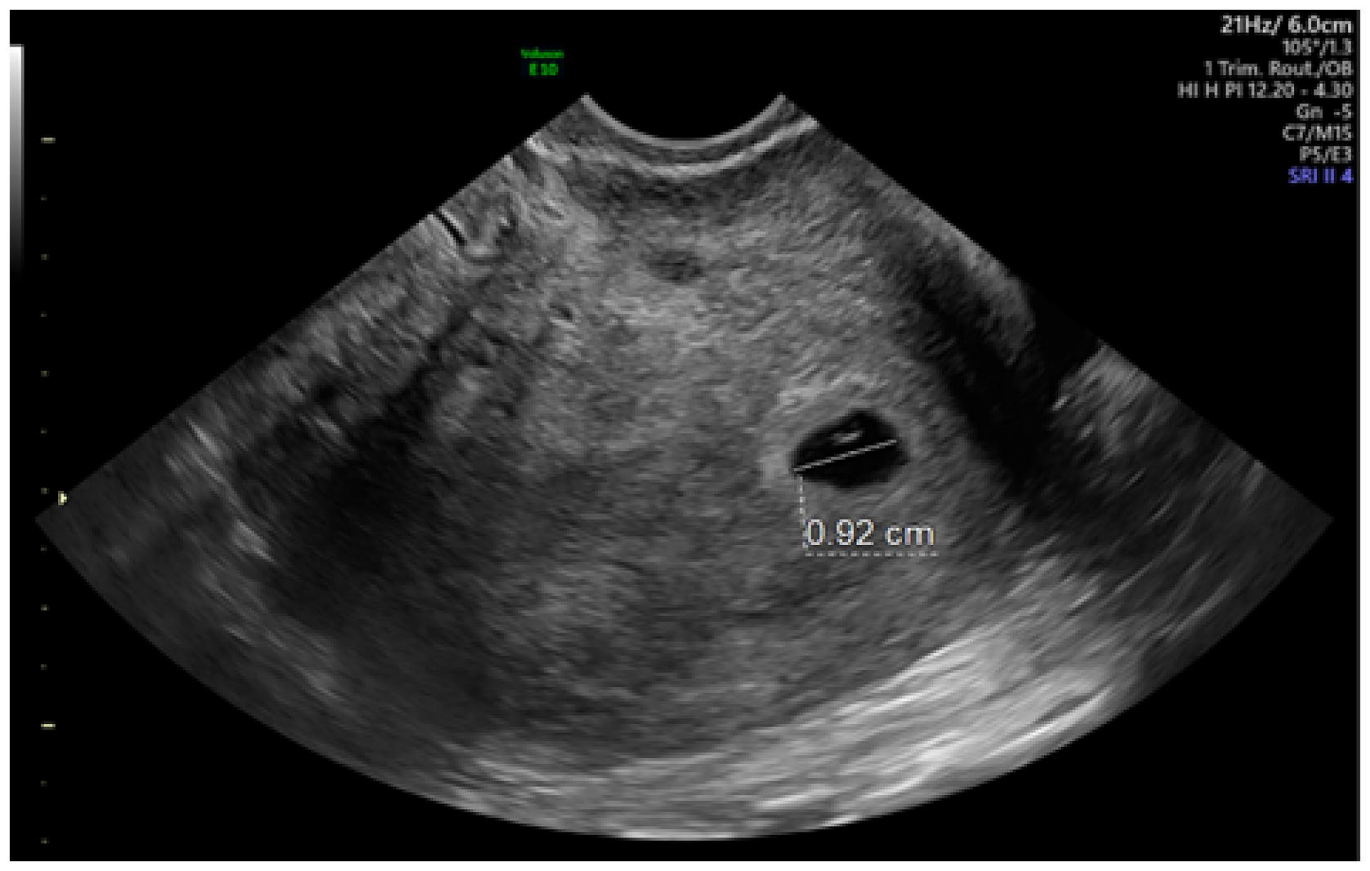
Transvaginal ultrasonographic image showing an intrauterine gestational sac with a gestational sac diameter of 9.2 mm and a yolk sac within the gestational sac, confirming an early intrauterine pregnancy. The gestational sac diameter corresponded to a gestational age of 5^+5^ weeks.

A 37-year-old woman with POI who had been managed with HRT for 2 years presented after a positive home urine pregnancy test performed because she failed to experience the expected withdrawal bleeding. Initial biochemical evaluation revealed a serum *β*-human chorionic gonadotropin (β-hCG) level of 310.8 mIU/mL, which appropriately doubled to 719.5 mIU/mL within 48 h. Subsequent transvaginal ultrasonography performed one week later confirmed the presence of an intrauterine gestational sac and a yolk sac, establishing the diagnosis of an early spontaneous intrauterine pregnancy (Figure 1).

**Figure 2:**
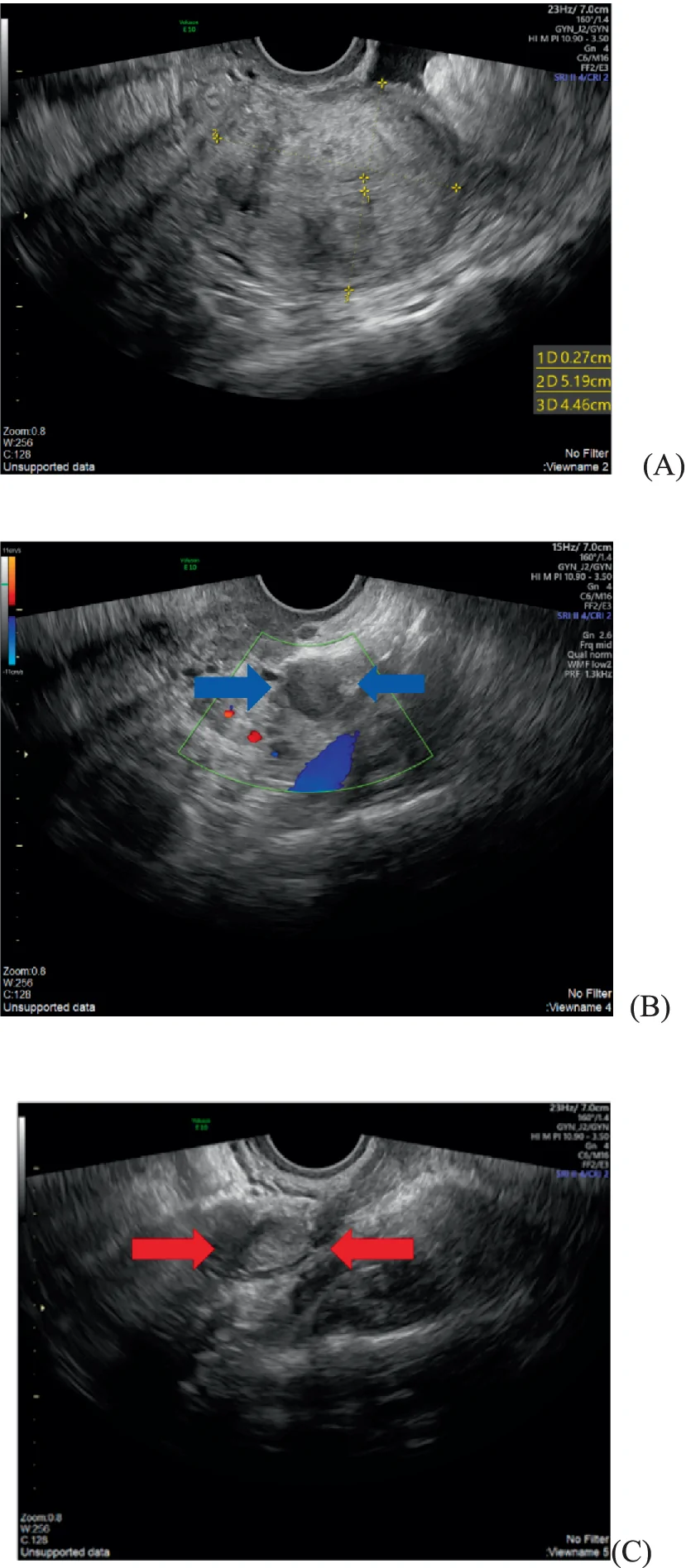
Transvaginal ultrasonography of the uterus demonstrates a measuring 5.19 cm × 4.46 cm diameter. The endometrium is thin and regular, with a measured thickness of 2.7 mm, showing no evidence of intracavitary pathology or focal thickening **(A)**. Ultrasound image of the left ovary (dimensions: 2.58 × 1.79 cm) revealing a hypoechoic lesion measuring 1.26 × 1.14 cm, highlighted by the blue arrow **(B)**. The right ovary measured 2.42 × 1.17 cm, as indicated by the red arrow **(C)**. No antral follicles are visualized in either ovary.

**Figure 3:**
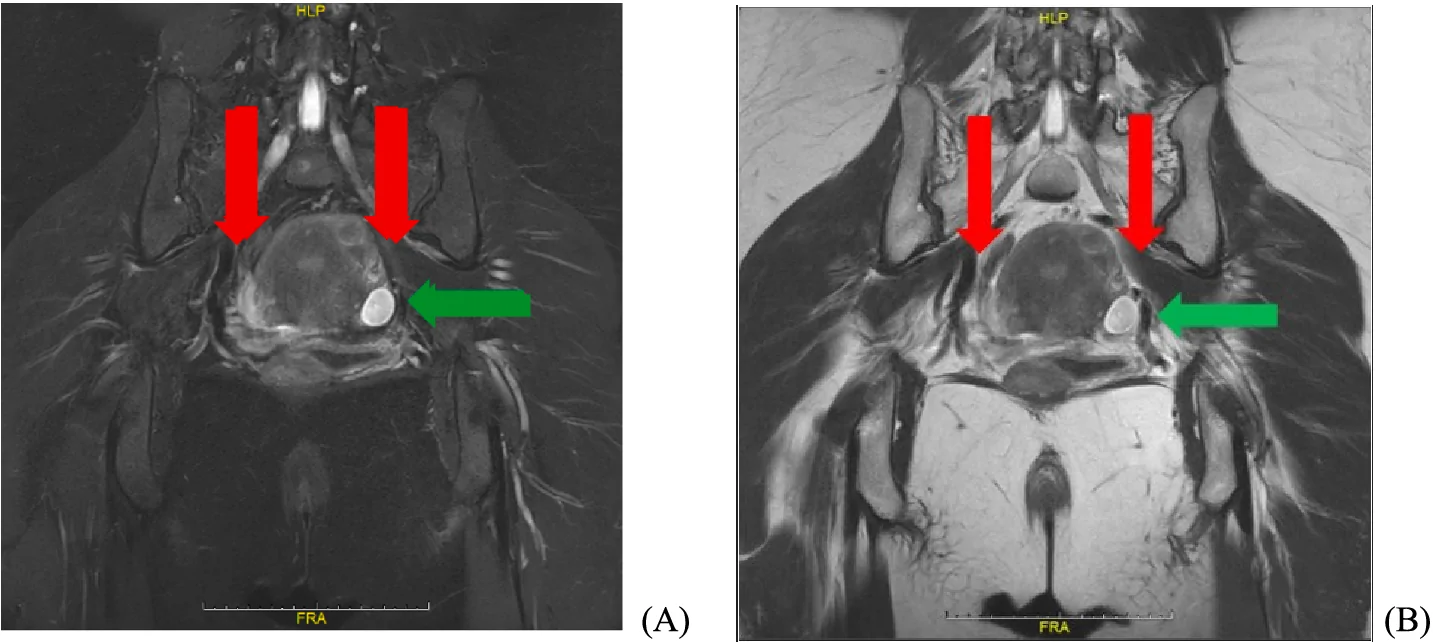
Coronal fat-suppressed T2-weighted **(A)** and T2-weighted **(B)** magnetic resonance image of the pelvis. The image clearly delineates a well-circumscribed, round, highly hyperintense cystic lesion measuring approximately 1.26 × 1.14 cm in the left adnexal region (green arrow). Both ovaries are poorly visualized (red arrow), making detailed assessment of antral follicles (AFs) impossible.

At the age of 36 years, the patient visited another hospital because of irregular menstrual cycles and vasomotor symptoms, including hot flushes, which had persisted for one year. Hormonal evaluation revealed a serum FSH level of 62.88 IU/L, an estradiol (E2) level of 15.0 pg./mL, and an anti-Müllerian hormone (AMH) level of 0.02 ng/mL. Her serum level of thyroid-stimulating hormone (TSH) and prolactin levels were 2.3 μIU/mL and 10.8 ng/mL, respectively. Based on these clinical and laboratory findings, she was diagnosed with POI. After the diagnosis of POI, hormone replacement therapy was initiated with Climen^®^ (estradiol valerate 2 mg/cyproterone acetate 1 mg). Because persistent abnormal uterine bleeding developed during the first 3 months of treatment, the medication was switched to Livial^®^ (tibolone). Despite this change, the bleeding continued. Dilatation and curettage were performed 5 months after the initiation of HRT. However, histopathological examination of the endometrial specimen revealed no significant abnormalities. She was subsequently referred to our hospital for further evaluation and management of abnormal uterine bleeding (AUB). At presentation to our hospital, the patient was a height of 165 cm and a body weight of 53 kg (body mass index [BMI], 19.46 kg/m^2^). She had discontinued HRT two months earlier and no vaginal bleeding was observed at the time of presentation. To evaluation her hormonal status and confirm the previous diagnosis, a repeat hormonal evaluation was performed 7 months after the initial testing. Hormonal evaluation at our hospital demonstrated FSH level of 66.55 IU/L, a luteinizing hormone (LH) level of 42.21 IU/L, E2 level of 12.62 pg./mL, and AMH level of 0.02 ng/mL. Serum levels of prolactin (8.4 ng/mL) and thyroid-stimulating hormone (TSH) (1.94 μIU/mL) were remarkably within normal limits. Based on these clinical and laboratory findings, the diagnosis of POI was confirmed. We prescribed Femoston 2/10^®^ (Estradiol hemihydrate 2 mg + Dydrogesterone 10 mg) for HRT, and she experienced no further episodes of AUB. Nevertheless, the patient had not been informed regarding the lack of contraceptive effect of the hormone treatment. Initial transvaginal ultrasonography revealed a uterus measuring 5.01 × 3.45 cm, with an endometrial thickness of 2.3 mm. The right and left ovaries measured 2.42 × 1.17 cm and 2.58 × 1.79 cm, respectively; notably, no antral follicles were detectable in either ovary. However, a 1.26 × 1.14 cm well-defined hypoechoic lesion was identified in the left ovary (Figure 2). To further characterize this adnexal mass, pelvic magnetic resonance imaging (MRI) was performed. The MRI confirmed poorly visualized ovaries bilaterally and demonstrated a lesion in the left ovary highly suggestive of an endometrioma (Figure 3).

She had undergone three laparoscopic procedures for ovarian endometrioma at the ages of 20, 21, and 30 years. Although the precise anatomical extent and operative details of each procedure could not be verified because detailed operative records were unavailable and the surgical history relied on the patient’s recollection, all procedures were reported to have involved both ovaries. To reduce the risk of endometriosis recurrence, the patient was prescribed Visanne^®^ (dienogest 2 mg) for two months after her second surgery at the age of 21 years, followed by treatment with the combined oral contraceptive (Mercilon^®^, ethinyl estradiol 0.02 mg/desogestrel 0.15 mg) for the subsequent eight years.

**Figure 4:**
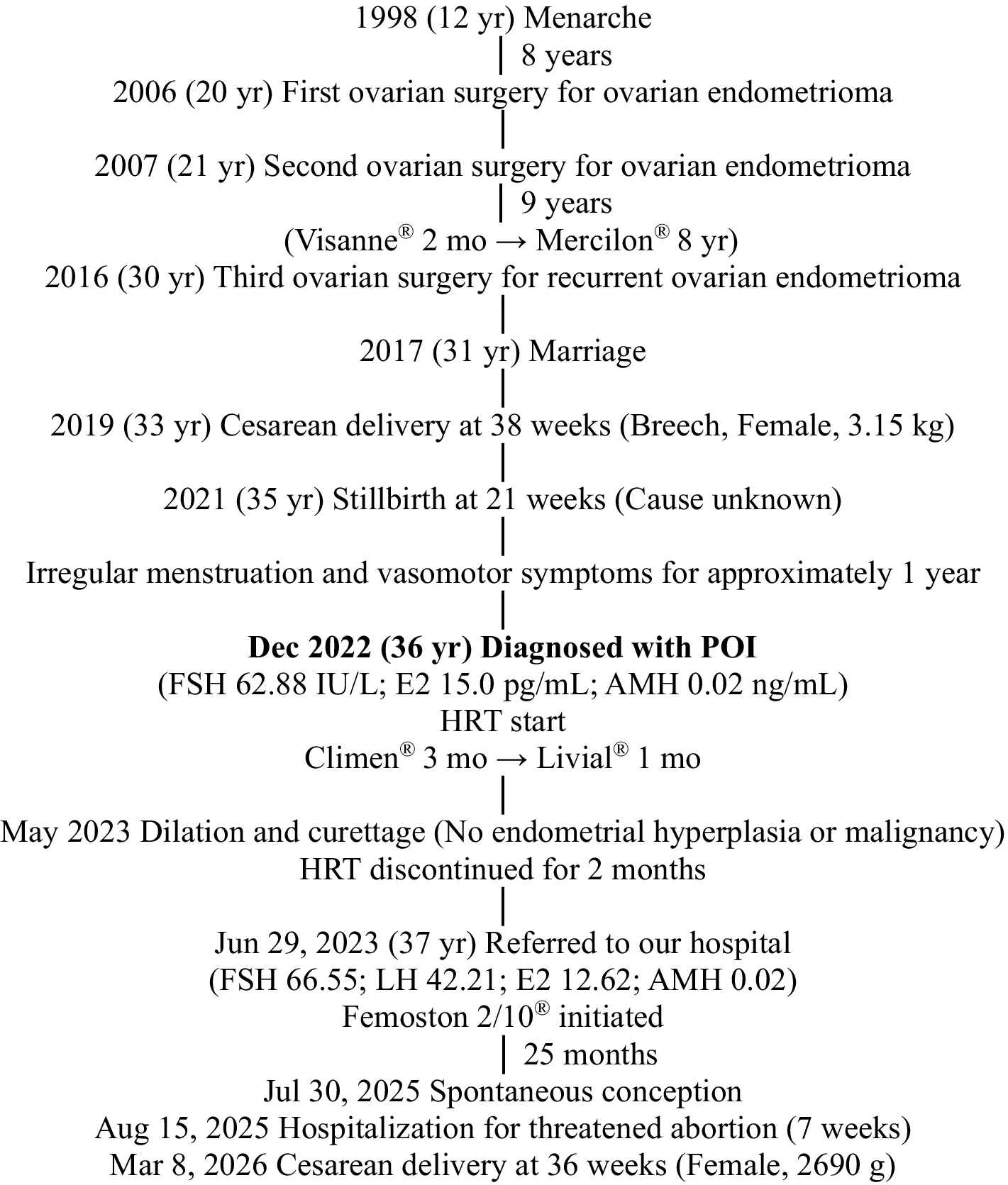
Chronological timeline of the patient’s clinical course. The timeline summarizes the major clinical events from menarche through repeated bilateral ovarian surgeries, the diagnosis of premature ovarian insufficiency, hormonal replacement therapy, spontaneous conception, pregnancy management, and delivery.

Regarding her obstetric history, the patient experienced menarche at age 12, followed by regular menstrual cycles. She married at age 31 and subsequently attempted to achieve pregnancy. Because of difficulty conceiving, she sought evaluation at a fertility clinic, where ovarian reserve testing suggested diminished ovarian reserve (DOR). At 33 years of age, she underwent a cesarean section at 38 weeks’ gestation for breech presentation and delivered a healthy female infant weighing 3.15 kg. Two years later, at the age of 35 years, she conceived again but experienced a stillbirth at 21 weeks’ gestation. The cause of the stillbirth remained undetermined because the parents declined further investigations, including fetal autopsy and placental biopsy. Although antiphospholipid syndrome (APS) was initially suspected at that time, subsequent diagnostic evaluations yielded negative results. As part of the evaluation, chromosomal analysis was performed for both parents and demonstrated normal karyotypes. The chronological clinical course of the patient is summarized in Figure 4.

**Figure 5:**
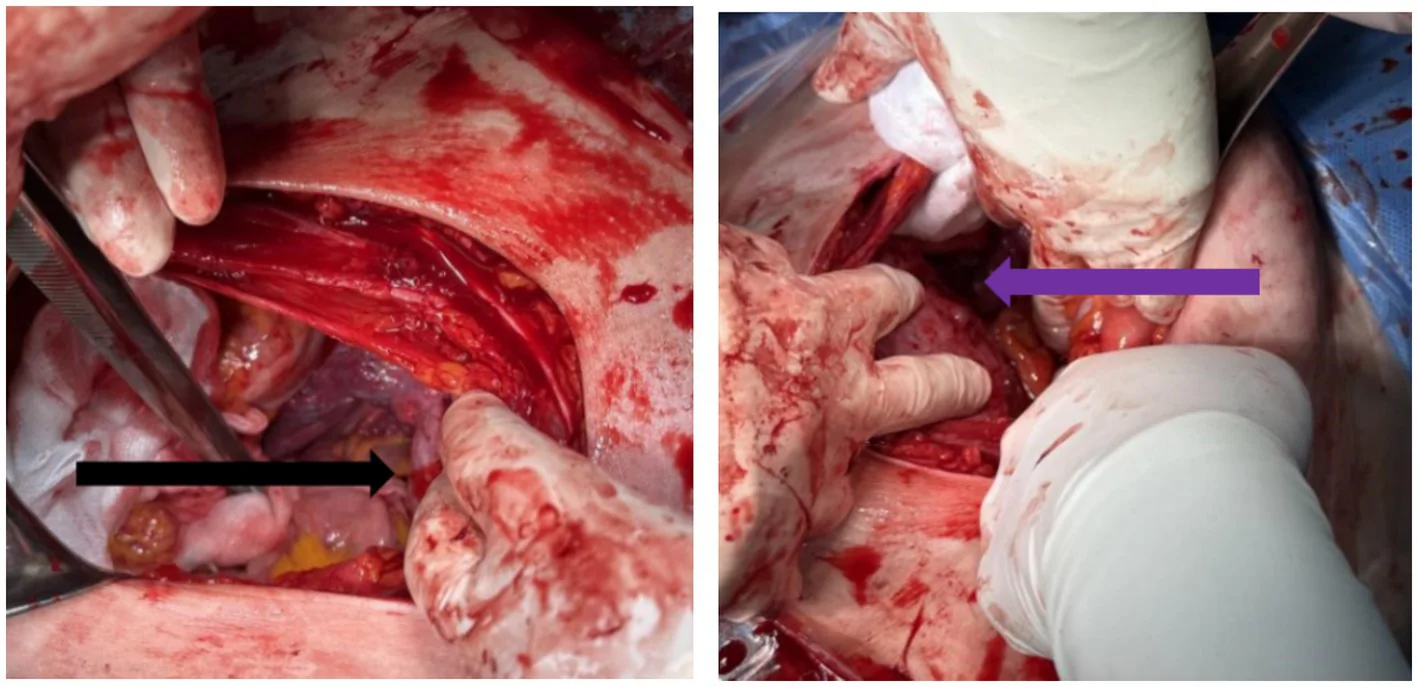
Intraoperative findings of the bilateral ovaries. Both ovaries were severely atrophic and extremely difficult to identify during the procedure. The anatomical locations of the right and left ovaries are indicated by black and purple arrows, respectively.

Subsequently, regular antenatal care was established. HRT was discontinued immediately upon confirmation of pregnancy. Given the potential for impaired ovarian function associated with POI, vaginal progesterone (Utrogestan^®^, 200 mg/day) was administered for luteal phase support until 12 weeks of gestation. At the time of pregnancy diagnosis, AMH level remained at 0.02 ng/mL. At 7 weeks of gestation, the patient presented to the emergency department with vaginal bleeding. Ultrasonographic evaluation revealed a viable fetus with detectable fetal heart tones (FHT) and a concomitant subchorionic hematoma. Under the diagnosis of threatened abortion, she was admitted for 2 days of bed rest and conservative management, and was subsequently discharged following the resolution of bleeding. Follow-up prenatal examinations were unremarkable. Although chromosomal screening was recommended due to advanced maternal age, the patient declined the procedure. A cesarean delivery was planned in the late preterm period due to maternal anxiety over stillbirth. After counseling the patient on the potential complications of preterm birth, and informed consent was obtained. A repeated cesarean section was performed at 36 weeks of gestation, resulting in the delivery of a healthy female infant weighing 2,690 g, with Apgar scores of 8 and 8 at 1 and 5 min, respectively. Due to a stable overall condition, including reassuring respiratory status, the neonate did not require admission to the neonatal intensive care unit (NICU). Following routine newborn care, the infant was discharged home together with patient. Intraoperatively, bilateral ovaries were noted to be markedly atrophic and poorly visualized (Figure 5). The postoperative course was uneventful, and she was successfully discharged on postoperative day 3. After discharge, the patient was followed up in the outpatient clinic at 2 and 8 weeks postoperatively. No surgical site infections or other complications were observed. At the 8-week visit, she reported no vasomotor symptoms, such as hot flashes, and noted that she was currently breastfeeding. After clinical counseling, it was decided to perform endocrine evaluation after the cessation of breastfeeding to determine the subsequent therapeutic strategy. For contraception, the couple decided that the husband would undergo a vasectomy.

## Discussion

POI has profound adverse effects on both reproductive function and overall health in young women. Therefore, early diagnosis, individualized management are essential to minimize long-term health risks and improve clinical outcomes. In addition, ovarian reserve should be carefully assessed and monitored not only in women diagnosed with POI but also in those at high risk of developing the condition. The measurement of ovarian size and follicle number using ultrasonography is a key approach to evaluating ovarian reserve. Although various criteria have been proposed for antral follicle count (AFC), an AFC of less than 5 to 10 is generally considered indicative of diminished ovarian reserve, while a count of 0 to 2 strongly suggests suspected POI (8). In recent years, AMH has emerged as an additional marker for assessing ovarian function, alongside conventional measures such as AFC and FSH levels. AMH is secreted by granulosa cells of growing follicles and is considered to reflect the size of the remaining follicular pool within the ovary. Because AMH levels decline before the onset of menopause and prior to a rise in FSH levels (9), it has been regarded as a useful biological marker for the early diagnosis and monitoring of declining ovarian reserve. While. Various cutoff values of AMH have been proposed as potential marker of POI, a threshold of 1.12 ng/mL (8 pmol/L) demonstrated a sensitivity of 85% and a specificity of 100% for diagnosing POI in a cohort study (10). In another large-scale study, using an AMH cutoff value of 0.25 ng/mL or less (1.78 pmol/L) resulted in a sensitivity and specificity of 92.46 and 90%, respectively (11). Nevertheless, the routine use of AMH for the diagnosis of menopause is currently not recommended.

Advancements in medical technology for diagnosing and treating malignancies in young women have led to an increasing trend in iatrogenic POI. While surgery for malignancies is well known to affect ovarian reserve, operations for benign conditions, such as the torsion of an ovarian cyst pedicle or ovarian endometriomas, are also highly detrimental to ovarian function. The severity of ovarian reserve depletion after ovarian endometriosis surgery depends significantly on the scope of endometriosis, cyst size, and the surgical techniques. Clinical factors including advanced age, elevated body mass index (BMI), shortened menstrual intervals, bilateral ovarian cysts, advanced surgical stage such as ASRM stage III/IV, repeated surgery for recurrent endometrioma, and total involvement of the pouch of Douglas are associated with a greater reduction in AMH levels following surgery, which increasing the risk of POI. Specifically, ovarian insufficiency is reported to occur in 2.4% of patients undergoing surgical removal of bilateral ovarian endometriomas (12). For these reasons, meticulous care is required during the surgical management of ovarian endometriomas to minimize iatrogenic ovarian damage. Several underlying mechanisms have been proposed to explain the post-operative decline in ovarian reserve, including the excessive removal of normal ovarian tissue, vascular damage induced by electrocoagulation, and subsequent autoimmune reactions triggered by surgery-induced severe local inflammation. Although ovarian reserve is influenced by the surgical technique used on the affected ovary, the specific hemostatic method implemented also yields varying degrees of impact; specifically, ovarian suturing with simple or barbed sutures has been reported as the most effective strategy for maintaining ovarian reserve compared to electrocoagulation, ultrasonic coagulation, and hemostatic matrices (13). Furthermore, the risk of developing POI following endometrioma surgery is heavily influenced by patient age. Ovarian insufficiency rates demonstrate an age-dependent increase, with surgical interventions performed at a younger age predicting a higher probability of subsequent ovarian insufficiency. When planning surgical interventions for these high-risk patients, clinicians must thoroughly counsel them regarding the potential risk of postoperative decline in ovarian function and provide comprehensive guidance on strategies to preserve and maintain their ovarian reserve. For women of reproductive age with endometriosis, fertility preservation should be considered before ovarian surgery, particularly in those who have not yet completed childbearing or are expected to have a poor reproductive prognosis. Among the available fertility preservation strategies, oocyte cryopreservation is currently regarded as the preferred option for patients with endometriosis. Oocyte cryopreservation offers several distinct advantages, as it does not further compromise the remaining ovarian reserve, is associated with minimal procedural morbidity, and does not require a male partner, making it the preferred fertility preservation strategy for post-pubertal women. Other available options include embryo and ovarian tissue cryopreservation; however, their clinical applicability in patients with endometriosis is more limited. Embryo cryopreservation requires sperm from a partner or donor and may raise ethical, legal, and psychosocial concerns. Ovarian tissue cryopreservation, although increasingly established for fertility preservation in oncology, is reserved for carefully selected patients with endometriosis because the procedure requires excision of healthy ovarian cortical tissue, which may further reduce the ovarian reserve.

HRT is essential for maintaining long-term health in patients with iatrogenic POI, similar to patients with idiopathic POI. Estrogen replacement therapy not only resolves diverse symptoms arising from estrogen deficiency but also delays or prevents subsequent chronic diseases, including osteoporosis, genitourinary syndrome of menopause (GSM), and cardiovascular disease. Therefore, current guidelines recommend initiating HRT immediately after a POI diagnosis and maintaining it until the expected age of natural menopause. Because iatrogenic POI arises from various causes, patients must undergo a thorough comprehensive evaluation before starting HRT to ensure there are no contraindications to hormone therapy. While multiple administration routes are available, transdermal delivery is considered the optimal approach for this patient population. An important clinical consideration is that these HRT regimens do not provide contraception, as spontaneous intermittent ovarian activity may occur in patients with POI. Therefore, alternative contraceptive methods should be recommended for those who do not desire pregnancy.

Fertility management represents another vital component in the care of patients with POI. When spontaneous pregnancy fails to occur, various infertility treatments, such as ovulation induction, are often attempted; however, no significant clinical differences have been observed among these modalities, and they are generally considered ineffective for this population. Under these circumstances, oocyte donation remains the most effective fertility option available. However, estrogen replacement therapy, administered over short or long periods, is documented to benefit patients with POI by stimulating follicle growth and potentially leading to spontaneous conception. The underlying mechanism is presumed to involve the positive impact of estrogen replacement, which downregulates gonadotropin secretion through a negative feedback loop, consequently stimulating follicular maturation. Spontaneous conception in women with POI has been reported predominantly among those receiving HRT. It has been reported that combining ovarian stimulation with hormone therapy yields markedly superior follicle growth rates and live births compared to hormone replacement alone. Furthermore, within the cohort under 35 years of age—comprising both idiopathic POI patients and those surgically treated for benign ovarian tumors—a duration of amenorrhea of less than 4 years coupled with *in vitro* fertilization–embryo transfer (IVF-ET) intervention resulted in a higher live birth rate. Consequently, integrating IVF-ET with hormone replacement represents a practical alternative that should be attempted prior to transitioning to oocyte donation (7). Poor pregnancy outcomes have also been reported in these patients. The ESHRE guideline on POI states that the obstetric risk following spontaneous pregnancy in women with idiopathic POI does not differ significantly from that of women with normal ovarian function. However, recent studies have demonstrated that POI is associated with an increased risk of early pregnancy loss across all modes of conception, including spontaneous conception, assisted reproductive technology (ART)-induced pregnancy, and oocyte donation.

This report has several limitations. First, because the patient was referred to our institution approximately two years before the spontaneous pregnancy occurred, much of her past medical history relied on the patient’s recollection rather than a comprehensive review of her prior medical records. Consequently, detailed information regarding the three previous surgeries for endometriosis was unavailable. Specifically, the exact surgical procedures performed, the size and laterality of the ovarian endometriomas, the extent of cyst excision and inadvertent ovarian tissue removal, and the detailed histopathological findings could not be fully verified. As a result, we were unable to precisely evaluate the cumulative impact of the repeated ovarian surgeries on ovarian reserve or to determine the extent to which each procedure contributed to the subsequent development of iatrogenic POI. Second, the diagnostic evaluation for POI was not exhaustive. In this case, genetic testing for the *FMR1* premutation and autoimmune investigations, including thyroid autoantibodies and 21-hydroxylase autoantibodies (21-OH Abs), were not performed. However, according to the 2024 ESHRE guideline, these investigations are recommended primarily for women with suspected non-iatrogenic or unexplained POI. Given this patient’s medical history of multiple bilateral ovarian surgeries for recurrent endometriosis, an iatrogenic etiology was considered the most plausible cause of her POI. Finally, as this is a single-case report, the findings should be interpreted with caution and may not be generalizable to all patients with iatrogenic POI.

Despite these limitations, this case has important clinical implications. Spontaneous pregnancy without ART is exceptionally rare in women with iatrogenic POI, particularly after repeated bilateral ovarian surgery for recurrent endometriosis. To the best of our knowledge, no closely comparable case has been reported. Previously published cases with similar clinical characteristics are summarized in Table 1 (14–19). Among the reported cases, one patient described as having iatrogenic POI had a serum FSH level of only 18.83 IU/L, which does not satisfy the currently accepted diagnostic criteria for POI. Furthermore, pregnancy in that case was achieved using assisted reproductive techniques involving ovarian stimulation with letrozole, rather than through spontaneous conception (15). Two other reports involved patients with idiopathic POI rather than iatrogenic POI (16, 17), and one of these pregnancies was achieved through oocyte donation (17). In addition, two reported cases involved POI resulting from gonadotoxic chemotherapy (14)and uterine artery embolization (18), respectively, representing etiologies distinct from the repeated ovarian surgeries for endometriosis observed in our patient. Another case described spontaneous pregnancy during hormone replacement therapy in a woman with autoimmune POI; however, serum FSH, estradiol, and AMH levels, were not provided, limiting the ability to confirm the diagnosis and compare the degree of ovarian insufficiency with our patient (19).

**Table 1:** Summary of previous reported cases of spontaneous pregnancy in women with premature.

Number (Reference)	Cause of POI	Age	FSH/E2 (IU/L/pg./mL)	AMH (ng/mL)	HRT	Spontaneous pregnancy	Duration of amenorrhea	Live birth
1 (14)	Chemotherapy	25	80/20	(−)	Yes	Yes	3 years	Yes
2 (15)	Chemotherapy	33	18.83/<10.0	0.1	No	No (FSH + letrozole)	5 years	Yes
3 (16)	Idiopathic	31	69.1/43.3	(−)	Yes	Yes	10 years	Yes
4 (17)	Idiopathic (salpingectomy d/t ectopic pregnancy)	26	62/10	(−)	No	No (oocyte donation)	17 months	Yes
5 (18)	Uterine artery embolization	40	25.2*	0.1	Yes	Yes	1 year	Yes
6 (19)	Autoimmue (antithyroid antibody)	26	Postmenopausal range/low level	(−)	Yes	Yes	12 years	Yes

Ovarian insufficiency.

PIO, premature ovarian insufficiency; FSH, follicle stimulating hormone; E2, estradiol 2; AMH, anti-mullerian hormone; HRT, hormone replacement therapy.

*Serum estradiol (E2) levels were not measured.

## Conclusion

We report a case of spontaneous pregnancy in a woman with iatrogenic POI following repeated bilateral ovarian surgeries for endometriosis. Despite biochemical evidence of ovarian insufficiency, she conceived naturally during hormone replacement therapy and delivered a healthy infant. This case suggests that intermittent ovarian function may rarely recover in some women with iatrogenic POI and underscores the importance of individualized counseling regarding the possibility of spontaneous pregnancy.

## Data Availability

The original contributions presented in the study are included in the article/supplementary material, further inquiries can be directed to the corresponding author.
